# The global distribution of fatal pesticide self-poisoning: Systematic review

**DOI:** 10.1186/1471-2458-7-357

**Published:** 2007-12-21

**Authors:** David Gunnell, Michael Eddleston, Michael R Phillips, Flemming Konradsen

**Affiliations:** 1 Department of Social Medicine, University of Bristol, Bristol, UK; 2 South Asian Clinical Toxicology Research Collaboration (SACTRC); 3 Scottish Poisons Information Bureau, University of Edinburgh, Edinburgh, UK; 4 Beijing Suicide Research and Prevention Center, Beijing Hui Long Guan Hospital, Beijing, China; 5 Department of Psychiatry, Columbia University, New York, USA; 6 Department of Epidemiology, Columbia University, New York, USA; 7 Department of International Health, University of Copenhagen, Copenhagen, Denmark

## Abstract

**Background:**

Evidence is accumulating that pesticide self-poisoning is one of the most commonly used methods of suicide worldwide, but the magnitude of the problem and the global distribution of these deaths is unknown.

**Methods:**

We have systematically reviewed the worldwide literature to estimate the number of pesticide suicides in each of the World Health Organisation's six regions and the global burden of fatal self-poisoning with pesticides. We used the following data sources: Medline, EMBASE and psycINFO (1990–2007), papers cited in publications retrieved, the worldwide web (using Google) and our personal collections of papers and books. Our aim was to identify papers enabling us to estimate the proportion of a country's suicides due to pesticide self-poisoning.

**Results:**

We conservatively estimate that there are 258,234 (plausible range 233,997 to 325,907) deaths from pesticide self-poisoning worldwide each year, accounting for 30% (range 27% to 37%) of suicides globally. Official data from India probably underestimate the incidence of suicides; applying evidence-based corrections to India's official data, our estimate for world suicides using pesticides increases to 371,594 (range 347,357 to 439,267). The proportion of all suicides using pesticides varies from 4% in the European Region to over 50% in the Western Pacific Region but this proportion is not concordant with the volume of pesticides sold in each region; it is the pattern of pesticide use and the toxicity of the products, not the quantity used, that influences the likelihood they will be used in acts of fatal self-harm.

**Conclusion:**

Pesticide self-poisoning accounts for about one-third of the world's suicides. Epidemiological and toxicological data suggest that many of these deaths might be prevented if (a) the use of pesticides most toxic to humans was restricted, (b) pesticides could be safely stored in rural communities, and (c) the accessibility and quality of care for poisoning could be improved.

## Background

The World Health Organisation (WHO) estimated that there were 873,000 suicides worldwide in 2002[1] which makes suicide a major cause of premature mortality globally. A central component of suicide prevention strategies is restricting access to lethal means [2]; this is because impulsive acts of self-harm in persons with a low intent to die may, nevertheless, be fatal if high-lethality methods are easily accessible [3,4]. It is therefore important to identify the most commonly used methods of suicide worldwide both to formulate appropriate strategies for restricting access to highly-lethal methods and to improve the ability of health care systems to effectively treat individuals who use these methods

Based on studies carried out in Sri Lanka, in 1984 Jeyaratnam estimated there were 220,000 pesticide related deaths every year worldwide, most of which were suicides [5]. Recent extrapolations of data from a few countries in Asia suggest that there may be 300,000 suicides by deliberate ingestion of pesticides annually in this region alone[6,7]. The WHO reports that pesticides are now the most common method of suicide worldwide[8].

The most likely explanation for the high numbers of pesticide suicides in developing countries is the high case fatality associated with pesticide ingestion compared to the relatively low case fatality of many of the substances commonly taken in acts of self-poisoning in the West. For example case fatality among persons admitted to hospital for treatment of self-poisoning in England and Wales is <0.5% [9] but in rural areas of Sri Lanka it is 7%[10]. Self-poisoning with some commonly used pesticides is particularly lethal – case-fatality following paraquat and aluminium phosphide ingestion is in excess of 70% [11,12].

To date efforts to estimate the international burden of pesticide suicides have been based on relatively crude extrapolations from studies carried out in one or two countries[5-7]. The aim of this paper is to systematically review the world literature on the use of pesticides for suicide and, using the data retrieved, to estimate the number of pesticide suicides worldwide and in each of the WHO's six regions. For the purpose of this review we define pesticides as chemical products used for the control of unwanted animals, plants and fungi – primarily rodenticides, insecticides, weedicides and fungicides.

## Methods

### Data sources

A systematic search of Medline, Psyc-INFO and Embase databases for papers published between 1990 and 2007 documenting the use of pesticides for fatal and non-fatal self-harm was supplemented by an internet-based search (using Google) for national and international data on: (a) estimated total suicides; (b) estimated suicides by pesticide poisoning and (c) national population estimates.

For our searches of Medline, PsycINFO and EMBASE we used the following search terms coded in all fields (af) for the years January 1990 – June 2007: (Pesticide? or insecticide? or rodenticide? or paraquat or organophosphate? or organophosphorus.af or agricultural.af or agrochemical?).af and (suicid??.af or (self and harm).af or parasuicide.af or (Self-Injurious and Behavior).af or ((self?.af or deliberate.af) and poison???.af)). We did not restrict our search to English language papers, but only reviewed papers in other languages if they had English language abstracts.

In our internet searches we particularly focused on identifying data for those countries in each of the six WHO regions that are likely to make the greatest contribution to the world burden of pesticide suicides because of their large populations: Nigeria and Ethiopia (Africa); United States of America, Brazil and Mexico (the Americas); Pakistan, Iran and Egypt (Eastern Mediterranean); The Russian Federation, Turkey and Germany (Europe); India, Indonesia, and Bangladesh (South East Asia); China and Japan (Western Pacific). We also retrieved potentially relevant publications cited in retrieved articles and searched our personal reference collections and books for potentially relevant material.

We also identified data on the use of pesticides for non-fatal acts of self-poisoning, but these data were not used to estimate suicides for each region.

### Data synthesis

Data from all relevant articles were abstracted by DG. Suicide data for the six WHO regions were obtained from the 2004 World Health Report [1]. In this report countries in each of the six WHO regions are categorised into five mortality strata depending on levels of child and adult mortality [Stratum A: Very low child, very low adult mortality; Stratum B: Low child, low adult mortality; Stratum C: Low child, high adult mortality; Stratum D: high child, high adult mortality; Stratum E: High child, very high adult mortality][1]. Estimates of the number of suicides within each stratum are recorded by WHO.

We assumed the proportion of all suicides due to pesticides in each country within a regional mortality stratum is similar, so we estimated the percentage of suicides due to pesticides within each mortality stratum for each of the six regions based on those countries for which we identified data in our searches. Where several sources of data are available for a single country we selected the most geographically comprehensive data, or if several such sources existed we used the most recent data. Where there were data for several countries within a stratum, we pooled these estimates weighting them according to the size of the populations (source: WHO World Health Report 2004[1]) of the contributing countries. Thus if data were available from four countries within a stratum, and those country's populations were 4.5 million, 3 million, 2 million and 0.5 million we gave a weight of 0.45 to the estimate from the largest country and only 0.05 to the estimate from the smallest country. Where data were available for only one country within a stratum, we calculated 95% confidence intervals around our estimate for that stratum based on the 95% confidence intervals for the estimate from the relevant country. Where data for several countries were used for our estimate, we used data from the country reporting the lowest proportion of pesticide suicides for the lower limit of our plausible range and that reporting the highest proportion for the upper limit. Where data on total pesticide suicides were given without a denominator for total suicides in that country, we obtained data on overall suicides from the WHO's Statistical Information System 108] for the relevant or closest years. For Jordan, the only country with pesticide suicide data in the Eastern Mediterranean-B stratum, no data on the total number of suicides (all methods) were available from WHO. In this stratum our estimates were derived by applying Jordan's overall pesticide suicide rate to the region's population and dividing this figure by WHO's estimate for the region's total number of suicides.

To estimate the global burden of pesticide suicides we summed total pesticide suicides for each regional mortality stratum. For the plausible range for the world total we summed totals for the lower and upper plausible estimates for each regional stratum.

In view of concerns about the reliability of national suicide data from India, we carried out a sensitivity analysis using literature-based estimates of India's suicide rate and of the proportion of suicides due to pesticides.

## Results

**Figure 1:**
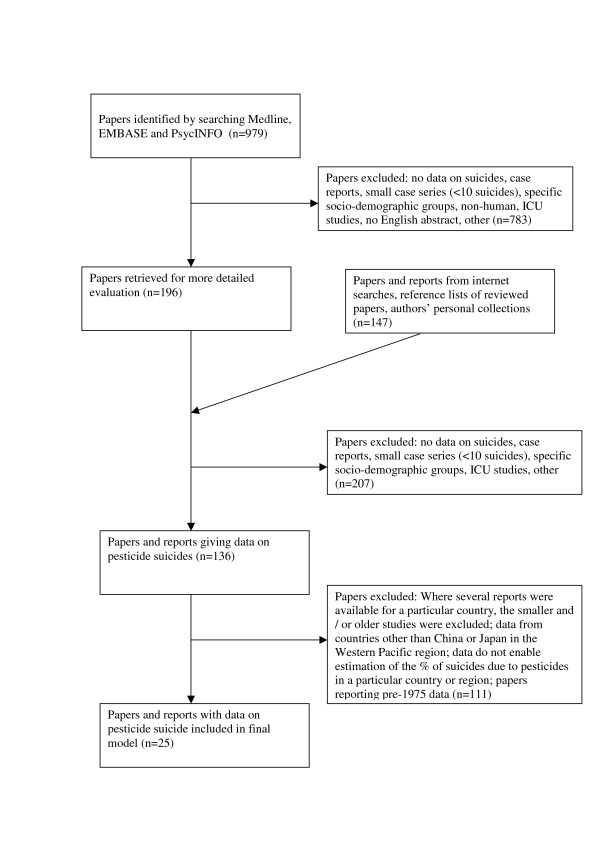
Flow diagram for data extraction.

Our final estimate of the global pesticide suicides is based on data extracted from 25 of the 343 papers and reports (Figure 1) retrieved for detailed evaluation. Table 1 presents summary data for overall and pesticide suicides for the mortality strata for each of WHO's six regions.

**Table 1:** Estimates and plausible ranges of pesticide suicides in each of WHO's six regions

**Region**	**Stratum**	**Population (millions) (source: [1])**	**Total suicides (source: [1])**	**Pesticide suicides (% of all suicides)**	**Plausible range of pesticide suicides**	**Sources**
Africa	D	311	15,000	4,950 (33%)	3,300 to 6, 900^†^	[22]
	E	361	19,000	2,850 (15%)	1,900 to 15,010	[23] [24] [25]
*Sub-total (Africa)*			*34,000*	*7,800 (22.9%)*	*5,200 to 21,910*	
The Americas	A	336	35,000	25 (0.07%)	14 to 35^†^	[26]
	B	445	26,000	2,860 (11%)	1,820 to 8,060	[27] [33] [36]
	D	74	2,000	220 (11%)	140 to 620	[27] [33] [36]
*Sub-total (the Americas)*			*63,000*	*3,105 (4.9%)*	*1,974 to 8,715*	
Eastern Mediterranean	B	143	9,000	629 (7%)	501 to 772^†^	[38]
	D	360	25,000	5,000 (20%)	4,000 to 6,250^†^	[41]
*Sub-total (E. Med.)*			*34,000*	*5,629 (16.5%)*	*4,501 to 7,022*	
Europe	A	415	48,000	2,400 (5%)	14 to 4,800	[46] [47] [48] [50]
	B	223	23,000	920 (4%)	18 to 1,610	[52] [53] [54] [55] [56]
	C	240	92,000	2,760 (3%)	1,840 to 2,760^†^	[57]
*Sub-total (Europe)*			*163,000*	*6,080 (3.7%)*	*1,872 to 9,170*	
South East Asia	B	298	37,000	9,250 (25%)	5,920 to 19,980	[62] [63]
	D	1,293	209,000	41,800 (20%)	41,800 to 62,700	Table 2 and [58]
*Sub-total (SE Asia)*			*246,000*	*51,050 (20.7%)*	*47,720 to 82,680*	
Western Pacific	A	155	35,000	1,050 (3%)	1,050 to 1,050	[68]
	B	1,562	296,000	183,520 (62%)	171,680 to 195,360	[70]
*Sub-total (W Pacific)*			*331,000*	*184,570 (55.8%)*	*172,730 to 196,410*	

**Overall Total**		**6,225**	**873,000**	**258,234 (30%)**	**233,997 (27%) to 325,907 (37%)**	

Total including more realistic estimate for total and pesticide suicides in SE Asia – D (n = 504,800)*			***1,181,200***	***371,594 (31%)***	***347,357 (29%) to 439,267 (37%)***	

*Assuming 30% of these are due to pesticides.

^†^Plausible range based on 95% confidence intervals around the proportion of suicides due to pesticides in the country whose data were used for estimate in this stratum

### Africa (Figure 2)

**Figure 2:**
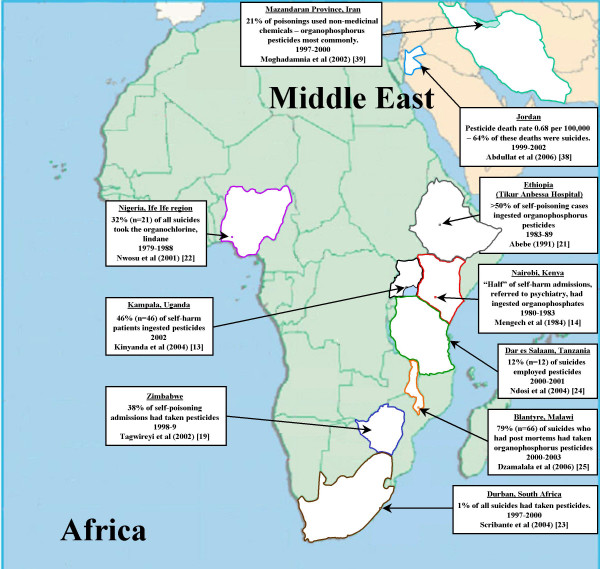
Pesticide suicides in Africa and the Middle East.

Data on suicide and the methods used for suicide on the African continent are sparse. For those countries where the substances taken in episodes of self-poisoning have been documented, there appears to be considerable variability in the contribution of pesticide poisoning. Data on hospital admissions (fatal and non-fatal) for self-poisoning indicate that pesticides were used in 46% of episodes in Kampala (Uganda) in 2002,[13] about 50% in Nairobi (Kenya, 1980–3) [14], 3%–30% in Nigeria in the 1970s–80s [15-17], about 40% in the major hospitals in Zimbabwe in the 1980s and 90s [18,19], and about 50% in Ethiopia in the early 1980s [20,21].

Our pesticide mortality estimate for Africa is based on data from four studies carried out in mainly urban regions within Nigeria (Stratum-B), South Africa (Stratum-E), Tanzania (Stratum-E), and Malawi (Stratum-E). These four countries comprise 32% of the region's population. Autopsy data for the Ile Ife region of Nigeria (1979–1988) suggest that a single pesticide (the organochlorine, lindane) accounted for 32% (n = 21) of the region's 65 suicides[22]. Only 1% (13/1018) of suicides in Durban, South Africa in 1997–2002[23]) employed pesticides; 12% (12/100) of suicides in Dar es Salaam, Tanzania in 2001–2 employed pesticides [24]; and 79% (66/84) of suicides in Malawi in 2000–3 employed pesticides[25]. Based on these data the weighted estimate of the proportion and number of suicides that employed pesticides in Africa is 22.9% and 7,800 (plausible range: from 5,200 to 21,910).

### The Americas (Figure 3)

**Figure 3:**
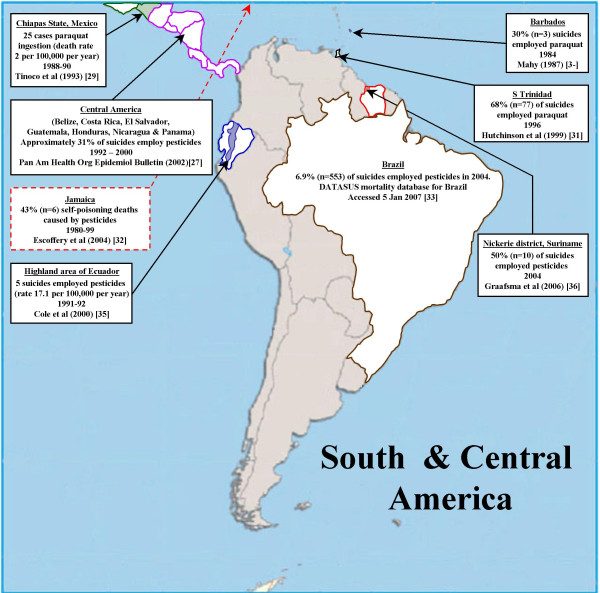
Pesticide suicides in Central and South America.

Pesticides are rarely used in acts of self-harm in North America. In the USA they were used in 87 suicides between 1995–1998 (approximately 22 per year)[26]. We found no data on the use of pesticides for suicide in Canada.

Grouped data for total pesticide deaths (suicide and non-suicide) were available for a number of Central American countries. Epidemiological surveillance data for Belize, Costa Rica, El Salvador, Guatemala, Honduras, Nicaragua and Panama indicate there were 748 pesticide related deaths in these countries in 2000[27]. If these data are correct, and the deaths are mainly suicides, then based on WHO suicide statistics for these countries (2,407 suicides based on an extrapolation of WHO data for 6 of these 7 countries), then pesticides account for 31% of suicides in this region. This figure contrasts with national suicide mortality data for Mexico, the largest country in Central America, indicating that poisoning (all substances) accounts for 5.9% of male and 27.5% of female suicides [28]; although in one area of rural Mexico death rates from paraquat poisoning in 1988–90 were around 2 per 100,000 per year [29]. In contrast, data from Barbados[30], Trinidad[31]and Jamaica[32]indicate pesticides account for a high proportion of suicides in those countries (see Figure 3).

Data for suicide using pesticides in South America are sparse. National statistics for the largest country in the region, Brazil (population 176 million) indicate there were 553 suicide deaths from pesticides in 2004 (accounting for approximately 6.9% of all suicides)[33]. Other data from Brazil suggest this figure may be an underestimate. In one study over half of all hospital admissions for self-harm to a General Hospital in Rio de Janeiro had taken pesticides[34]. Data for rural areas of Ecuador (5 pesticide suicides) [35] and Suriname (10 out of 20 suicides employed pesticides)[36] indicate that a high proportion of suicides in these areas employ pesticides. Jors *et al.*[37] cite an MSc thesis indicating that pesticides are the most commonly used method for self-harm in La Paz, Bolivia.

Our estimates of pesticide suicide in the Americas are based on data from the USA (stratum A), Brazil (stratum B), Suriname (stratum B) and the Central American Isthmus (stratum B), countries comprising 59% of the population of the region [26,33,36,27]. We had no specific data for pesticide suicides in countries in Americas stratum-D, but the data from the Central American Isthmus [27] included two stratum-D countries (Guatemala and Nicaragua) so we used data from Americas stratum-B for our estimates for stratum-D. The weighted proportion and number of suicides that employ pesticides in the Americas is 4.9% and 3,105 (plausible range: 1,974 to 8,715).

### Eastern Mediterranean Region (EMR) (Figure 2)

Few publications have presented data on suicide by pesticide poisoning in Eastern Mediterranean countries, where the WHO estimates that there are 34,000 suicides annually. In Jordan (population 5.1 million 1999–2002) – the only country in the EMR-B stratum with national pesticide suicide data – there were 90 pesticide suicides between 1999–2002, resulting in an estimated suicide rate by pesticide ingestion of 0.44 per 100,000[38]. Applying these rates to the population of EMR-B gives an estimated 629 suicides annually for this stratum i.e. 7% of all suicides. Data for poisoning (deliberate and accidental) in Iran, the most populous country in the EMR-B stratum, support the view that pesticides are likely to make an important contribution to mortality from suicide in that country [39,40].

Pesticide suicide data for the EMR-D stratum are only available for Pakistan, which comprises approximately 40% of the population of this stratum. The only national source of data on suicide methods identified was a review of newspaper reports based on police inquiries in Pakistan 1996–7 [41]: poisons were used in 39% of the 306 reported suicides and the authors state that organophosphorus pesticides and other agricultural chemicals were the main poisons used. This assertion is supported by findings from the Aga Khan University Hospital in Karachi, Pakistan indicating that between 1989–1995 organophosphorus pesticide poisoning accounted for 21% of self-harm admissions[42]. But lower estimates come from a series of 98 suicides in Faisalabad, Pakistan in 1998–2001 in which self-poisoning (all poisons) accounted for only 15% of deaths [43]. The only data we identified for Egypt came from a study of 200 episodes of hospital-presenting self-harm in Cairo in 1979: 10% of the episodes used poisons and disinfectants; organophosphates were the most frequently used poisons [44].

Our model estimates of pesticide suicides in the EMR were based on data from Jordan (stratum B)[38] and Pakistan (stratum D) [41] alone, countries comprising 31% of the population of the region. We assumed half of the poisoning suicides in Pakistan were due to pesticides and so estimated that 20% of suicides in EMR stratum-D were the result of pesticide ingestion. Overall in the Eastern Mediterranean region the weighted estimate of the proportion and number of pesticide suicides is 16.5% and 5,629. Based on confidence intervals around data for Jordan and Pakistan the plausible range for this estimate is 4,501 to 7,022.

### Europe (Figure 4)

**Figure 4:**
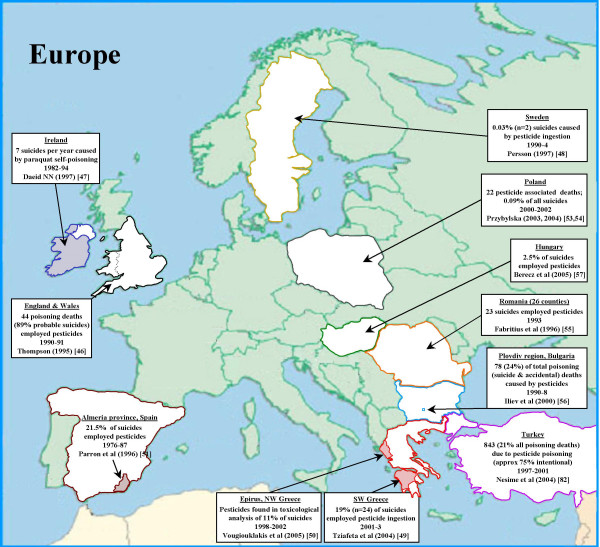
Pesticide suicides in Europe.

Pesticides are infrequently used in acts of self-harm in Europe. The WHO multi-centre study of self-harm in Europe (1982–1993) found that pesticides and agricultural chemicals were used in only 3% of all episodes reported by the 14 centres [45]. Hungary was an exception to this pattern; in the town of Szeged around 17% of self-harm episodes involved pesticides [45].

Few publications present national data on the use of pesticides for suicide in Europe. We found no data for either the largest country in Europe – the Russian Federation (population 144 million) or the second largest country, Germany (population: 82 million). Our estimates for the Europe-A stratum were based on data for England & Wales, Ireland, Sweden, Greece and Spain [46-51]; the Europe-B stratum on data from Turkey [52], Poland [53,54], Romania[55], and Bulgaria [56]; and stratum-C on data from Hungary [57].

For Europe-A stratum there were estimates of pesticide suicides from rural areas of Spain[51] and Greece [50,49]; to avoid placing too much weight on rural areas, we used the lower estimate – 10% (9/90) based on data from north west Greece [50]. National data from England & Wales, Ireland and Sweden suggest a lower incidence in countries within this stratum. In England and Wales 0.5% (n = 39) of the approximately 7,843 suicides in 1990–1 were pesticide ingestions[46]. Data for Ireland are restricted to suicide by paraquat ingestion in the agricultural sector; at least 2.0% (n = 82) of the approximately 3860 suicides in Ireland between 1982–1994 took paraquat [47]. Two of the 7006 (0.03%) suicides in Sweden 1990–1994 employed pesticides [48].

For Europe-B, data from the Trakya region of Turkey (1994–2004) suggest that 7% (10/137) of suicides employed pesticides [52]. The proportion is lower in Romania [55] where 1% (104/9,660) of suicides between 1990–1993 employed pesticides. Data from a region with approximately 8.8% of Bulgaria's population suggests that pesticides accounted for 78 poisoning deaths (suicide and accident) between 1990–98 (9 per year). Extrapolating these data to the whole of Bulgaria we estimate that 6.8% of Bulgaria's 12,990 suicides involve pesticide ingestion. Data from Poland for 2000–2002 indicate there were 22 pesticide-associated deaths amongst the country's 24,895 suicides over this period (0.09%)[53,54].

Data from Hungary (Stratum-C) indicate that around 2% (75/2979) of suicides employ pesticides [57].

Our estimate for Europe is based on data from countries comprising approximately 30% of the region's population. The weighted estimate for the proportion and annual number of pesticide suicides in Europe is 3.7% and 6,080 (plausible range 1,872 to 9,170).

### South East Asia (Figure 5)

**Figure 5:**
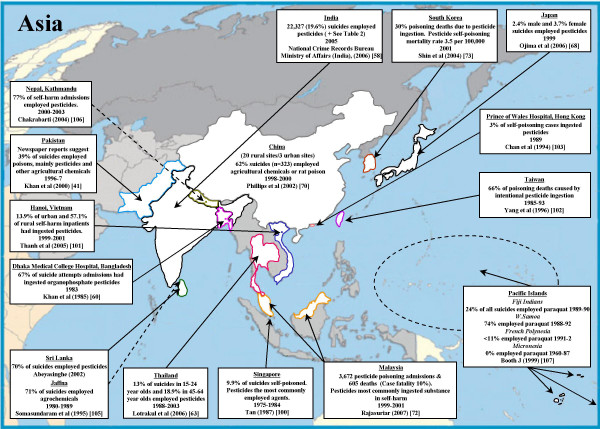
Pesticide suicides in South East Asia and Western Pacific regions.

Estimates for pesticide suicides in South East Asia are dominated by patterns of suicide in India (SE Asia mortality stratum D), whose population (1,050 million) accounts for 81% of the 1,293 million people living in stratum-D of the region. Official data for 2005 suggest that 19.6% (n = 22,327) of India's 113,914 officially recorded suicides were self-poisonings with insecticides[58]. A particular feature of self-poisoning in northern India is the frequent ingestion of aluminium phosphide, a fumigant used to protect grain stores, with an associated case fatality in excess of 70% [12]. The WHO estimate that 55% of suicides in Bangladesh (SE Asia-D), the third most populous country in this region (population 144 million), are by self-poisoning[59]. Data from other sources indicate that a large proportion of these fatal poisonings are pesticide ingestions[60,61]. Our estimates for stratum-D are based on official suicide statistics from India[58] and, using data presented in Table 2, an estimated range in the proportion of suicides that employ pesticides in India of 20% to 30%.

**Table 2:** Estimates of suicide rates and the contribution of pesticides to suicide in India

**Author**	**Years covered (no. suicides)**	**Setting**	**Estimated total (all methods) suicide rate**	**Estimates of the proportion of suicides (or episodes of self-harm) due to pesticides**
Nandi *et al.* [89]	1976–7 (n = 101)	Districts in West Bengal (rural)	Daspur area: 29 per 100,000	58% due to endrin (a pesticide)
			Chandrakona area: 5 per 100,000	37% due to endrin
Bannerjee *et al.* [90]	1978 (n = 58)	Villages in Deganga, West Bengal (rural)	43 per 100,000	93% suicides due to self-poisoning ("almost exclusively)" organophosphorus pesticides
Shukla *et al.* [91]	1986–7 (n = 187)	Jhansi City, Uttar Pradesh (urban)	29 per 100,000	10% (insecticides and rat poison).
Bhatia *et al.* [92]	Not stated (n = 55)	Delhi (urban and rural areas)	-	Only 13% of suicides had self-poisoned (all substances)
Joseph *et al.* [64]	1994–9 (n = 609)	Villages in Kaniyambadi (rural), Tamil Nadu	95 per 100,000	45% self-poisoning (all substances). 40% of the suicides in 15–19 year olds used pesticides in 1992–2001 [93]
Lalwani *et al.* [94]	1991–2000 (n = 222)	New Delhi (urban)	-	10–18 year olds: poisoning accounted for 49% of male and 37% female suicides. Pesticides were commonest poisons recorded.
Gururaj *et al.* [95]	2001–2 (n = 269)	Bangalore (urban)	-	28% (male) and 19% (female) suicides were self-poisoning (all substances).
Kumar *et al.* [96]	1994–2004 (n = 441)	Mannipal (rural)	-	>55% insecticides
Prasad *et al.* [65]	2000–2002 (n = 306)	Villages in Kaniyambadi (rural), Tamil Nadu	92 per 100,000	Organophosphorus pesticides accounted for 40.5% of suicides
Mohanty *et al.* [97]	2000–2003 (n = 588)	Berhampur (rural and urban)	-	30.6% of all suicides were self-poisoning (>70% used pesticides)
Bose *et al.* [66]	1998–2004 (n = 638)	Villages in Kaniyambadi (rural), Tamil Nadu	82 per 100,000	40% poisoning (majority pesticides)
Sharma *et al.* [98]	1996–2005 (n = 1421)	Chandigarh (rural and urban)	-	Aluminium phosphide accounts for 24% of all suicides; organophosphorus and organochlorine products 10%
Gajalakshmi *et al.* [67]	1997–8 (n = 3,249)	Villpuram district (rural), Tamil Nadu	62 per 100,000	53% self-poisoning ("generally involved agrochemicals")
Kumar P *et al.* [99]	2001 and 2005 (n = 200)	Six districts in rural Punjab	12.4 per 100,000 (2001) 13.1 per 100,000 (2005)	Pesticide/poison used in 77% (154/200) suicides studied.

Reasonable estimate of Indian suicide rate from these data: 40 per 100,000 (lower limit: 10 per 100,000 Indian Police Statistics [58]); upper limit 80 per 100,000 Tamil Nadu [64] [65] [66] [67].

The only countries in the SE Asia-B stratum with data on the proportion of suicides due to pesticides are Sri Lanka and Thailand. National mortality data from Sri Lanka indicate that 54% of suicides in that country were by pesticide ingestion in 2005 [62]. National data from Thailand (1998–2003) indicate that approximately 16% of suicides died from agricultural chemical poisoning[63].

Combining the data described above, the weighted estimate for the proportion and annual total of pesticide suicides in this region is 20.7% and 51,050, with a plausible range of 47,720 to 82,680. This estimate is based on data from countries comprising 71% of the population of the region.

The official suicide rate for India (10.3 per 100,000 in 2005[58]) is thought to be an under-estimate. Detailed studies in several regions (Table 2) suggest that India's suicide rates may be as high as 40 per 100,000 and that 30% or more of these deaths are due to pesticide self-poisoning. The studies reporting the highest suicide rates within India (>60 per 100,000) were carried out in rural Tamil Nadu [64-67]. Rates from these studies are three times higher than the official figures for Tamil Nadu[58]. Whilst some of the discrepancies between official suicide rates and those identified in local studies may be due to urban-rural differences in suicide incidence, police data suggest that around 90% of suicides in India occur outside cities[58]. It therefore seems appropriate to give more weight to estimates derived from rural populations. Using these data we estimate that there may be up to 420,000 suicides in India – 126,000 from pesticide self-poisoning. Applying the estimated suicides rates and proportion of pesticide suicides derived from Table 2 to the whole population of SE Asia D (1,293 million) yields a total of 517,200 suicides (i.e. an additional 308,200 above the official estimate of 209,000) and 155,160 pesticide suicides (i.e. an additional 113,360 pesticide suicides). These figures result in a revised estimate of pesticide suicides in this region of 164,410.

### Western Pacific Region (Figure 5)

Japan's population (127 million) comprises over 80% of the people in the WPR-A stratum and China's population (1,302 million) comprises over 80% of the WPR-B stratum. Estimates of pesticide suicides for these two countries alone were used to derive figures for the Western pacific region and sensitivity analyses were based on 95% confidence intervals around estimates of the proportion of suicides due to pesticides. Japanese national data indicate that 2.4% of the 22,402 male suicides and 3.7% of the 9,011 female suicides in 1999 were due to pesticide self-poisoning [68]; 3% of all suicides were due to pesticides. Applying this proportion to the WPR-A stratum we estimate that 1050 suicides are due to pesticide poisoning.

Pesticides are commonly taken in acts of self-poisoning in China. A study carried out in Northern China indicated that 28% of 14,771 emergency room visits for self-harm were due to pesticide ingestion[69]. A detailed study of a representative sample of 519 suicides from 23 sites in China (20 rural/3 urban) in 1998–2000 [70] found that 62% (323/519; 95%CI 58% to 66%) were the result of self-poisoning with agricultural chemicals or rat poison. Such high use of pesticides in acts of self-harm is confirmed by studies in Malaysia, where in one region pesticides accounted for >90% of suicides between 1973–84[71]. Recently published national data for Malaysia show that pesticides accounted for 605 deaths (mostly self-inflicted) amongst people admitted to hospital for poisoning between 1999–2001[72]. In South Korea over 20% of all suicides are due to self-poisoning with pesticides [73]. Using data from Japan (Stratum A) and China (Stratum B) our estimate of the proportion and number of pesticide suicide deaths in the Western Pacific Region is 55.8% and 184,570, with a plausible range of 172,730 to 196,410.

### World total

Combining data from all six regions our best estimate of the annual number of pesticide suicides worldwide is 258,234 with a plausible range of 233,997 to 325,907. This accounts for 30% (range 27% to 37%) of all suicides globally. If higher estimates of suicides in India are included in the model the total annual number of world suicides increases from 873,000 to 1,181,200, the estimated number due to pesticides becomes 371,594 (347,357 to 439,267) and the proportion of global suicides due to pesticide ingestion becomes 31% (29% to 37%).

## Discussion

### Main findings

We conservatively estimate that approximately one third (n = 258,234) of the world's 873,000 suicides in 2002 were caused by pesticide ingestion. Our estimate may well be too low, since it is influenced by the reliability of official suicide statistics for India. Recent detailed studies of rural India suggest that the true number of suicides may be 2–3 fold higher than official estimates and concentrated in rural areas where pesticide poisoning is common[67,64] a pattern that is also found in China [74].

The estimated proportion of suicides attributable to pesticide self-poisoning varies considerably across the WHO's six regions: in Europe we estimate 3.7% of suicides employ pesticides, the Americas: 4.9%, Eastern Mediterranean: 16.5%, Africa: 22.9%, South East Asia: 20.7% and Western Pacific: 55.8%.

Whilst our estimate of pesticide deaths is similar to that of 300,000 given in recent reviews [6,7], this is the first time an attempt has been made to estimate the number of pesticide self-poisoning using data from all six WHO regions. Our review indicates that previous crude extrapolations based on data from a limited number of countries have provided a relatively robust estimate of the extent of the problem. Pesticide poisoning and hanging are the two most commonly used methods of suicide worldwide, though precise data on their relative frequency are lacking. An important difference between suicides using these two methods is that deaths from pesticide self-poisoning are considerably more amenable than those from hanging to prevention by both restricting access to pesticides and improving the medical management of pesticide poisoning.

The number of deaths from pesticide self-poisoning under-estimates the true burden on health services of pesticide related self-harm[75]. Acts of pesticide self-poisoning are associated with a case fatality of between 1% and 70%, depending on the particular pesticide taken. Paraquat and aluminium phosphide self-poisoning have case-fatality in excess of 70%[11,12] whereas case fatality for the organophosphorus (OP) insecticides dimethoate and chlorpyrifos are 23% and 8% respectively [76]. Based on the crude assumption that overall case fatality following pesticide self-poisoning worldwide is between 10% (based on estimated case fatality in China, personal communication Michael Phillips) and 20% (based on data from India [30% case fatality, Table 2] and Malaysia 16%[72]), our conservative estimate of 258,234 pesticide deaths arise from between 1,291,170 and 2,582,340 episodes of pesticide self-poisoning annually.

OP insecticides appear to be the most commonly ingested pesticides in rural Asia, accounting for around two thirds of cases[77]. Depending on the particular OP ingested, around 20–30% of patients require intubation [78,79]. Two-thirds of intubated patients will be intubated for a median of 45 hours post admission. One third of intubated patients will develop late respiratory failure (intermediate syndrome) requiring intubation for a median of 284 hours [79]. As a result, across rural Asia, intensive care units are often filled with OP poisoned patients on ventilators, preventing the admission of other acutely ill patients [80,75]. Relatively few (<10%) of those poisoned with other pesticides require intubation. Patients ill with paraquat or aluminium phosphide poisoning are usually not intubated or admitted to intensive care because of their poor prognosis. These figures allow us to generate a crude estimate of person days of ventilation required each year of 1,147,000 to 2,294,000 days based on the high and low estimates of the number of episodes of pesticide self-poisoning (see Additional file 1 for details). This would require the constant use of 3,140 to 6,280 ventilators worldwide solely for managing self-poisoning with pesticides. But ventilators are not available in many parts of the developing world so lack of access to ventilators makes a substantial contribution to deaths from pesticide ingestion. Furthermore, the use of ventilators, where these are available, for cases of pesticide poisoning will compromise the capacity of hospitals to manage other life threatening conditions.

The global distribution of fatal pesticide self-poisoning does not mirror pesticide sales. The largest sales (29% of the world market) are in Europe [81] which accounted for only 2% (6,080/258,234) of pesticide suicides (Table 1). In contrast, Asia (comprising the Western Pacific and SE Asia regions in Table 1) accounts for approximately 25% of the world market [81] but 91% (235,620/258,234) of deaths [81]. This is probably due to the different agricultural practices in these regions – the large number of small holders practising agriculture in Asia allows easy access to pesticides, while the very restricted number of people working the land in Western countries means that few people are able to obtain pesticides in the quantities and strength that farmers use. Of note, the greatest market growth in pesticide sales is in Africa and the Middle East with a 9.1% rise in sales between 2004 and 2005[81] – so there may be a corresponding growth in pesticide self-harm in these regions in years to come.

### Limitations

The main limitation of this review is the absence of good quality cause-specific suicide mortality data for a number of the world's largest countries – most notably Indonesia, Iran, Russia, Germany and Ethiopia. Missing data from Europe are less problematic than data from Africa and the Eastern Mediterranean, as estimates for these latter regions are based on particularly limited data. Nevertheless, less than 10% of the world's suicides come from these areas. The availability of better quality data from these regions is therefore unlikely to greatly affect our estimates, unless the overall number of suicides in these regions has been grossly under-estimated. Our figures might be further refined through requests for pesticide mortality data from individual governments and hand searches of the grey literature.

The second limitation is in relation to the likely poor quality of India's suicide mortality data. India is the second largest country in the world, contributing almost 20% to the world's population. In a sensitivity analysis we used plausible estimates for suicide rates and the contribution of pesticides to suicide in India. This analysis resulted in an increase of our estimate of pesticide deaths to 371,594 – an increase of around 110,000 deaths. Obtaining nationally representative data on suicide rates, and the contribution of pesticide suicides to suicides in India, should be a priority for health funding agencies over the next decade. Poor quality data from other parts of the world are also likely to result in under-estimations. This is particularly the case as most pesticide suicides occur in rural (farming) districts where the quality of suicide data collection is likely to be poorer than in urban locations. Thirdly, we have no strong evidence that the proportion of pesticide suicides in countries within WHO mortality strata for each region is the same; mortality patterns are a crude marker of agricultural practices and the availability of toxic pesticides. Fourthly, in some instances the data obtained from the literature predated WHO's 2002 estimate of world suicides by up to 20 years e.g. figures for Nigeria were based on data for 1979–1988[22] and for Ireland for 1982–1994 [47]. Changes in the use of pesticides for self-harm may have resulted in us under- or over-estimating pesticide suicides within particular strata. Lastly, some of our estimates for pesticide suicides are based on combined accidental and suicide deaths; but where such deaths are separately categorised, the great majority of pesticide deaths are suicides so it is unlikely that conflating intentional and unintentional pesticide deaths in these countries would substantial alter the overall results [82,38].

### Policy options for reducing pesticide suicides

Pleas for national and international action to restrict the sale of pesticides to reduce their impact on human health date back over 40 years. In 1966 Ganapathi and colleagues wrote in the Journal of the Indian Medical Association "The present authors plead for a restriction in the sale of such lethal agents. It is hoped that a considerable number of young lives could then be saved from such a measure.... [83]."

In 2005 WHO launched a global initiative to tackle the burden of pesticide suicides[84]. Inactivity prior to this is likely to reflect the tensions between the perceived benefits of pesticides in increasing crop yields in low-income countries, where under-nutrition is an important contributor to poor health, and concern about the health effects of excessive exposure. Over the years the pesticide industry has responded to safety concerns with a number of initiatives to reduce the global death toll from pesticide poisoning. However, the pesticide industry is highly profitable – global sales of pesticides amounted to $31 billion in 2005[81] - and there are clear conflicts of interest where such initiatives may compromise profits. Industry led initiatives are to be welcomed, but national and international health policy makers should recognise that they may not focus on those aspects of the problem most likely to reduce mortality [85].

Restricting access to the most toxic pesticides is of paramount importance to reduce the number of severe self-poisoning episodes[86]. National policies and systems for enforcement need to be put in place immediately to phase out WHO Class I and the most toxic Class II pesticides. Likewise, in the longer-term, cost-effective alternatives have to be made available to farmers to reduce the overall use of pesticides. Further investments are needed to promote the safe storage of pesticides at community level. To further reduce the case-fatality from pesticide self-harm investments are needed to improve quality, affordability and accessibility of health care close to the affected communities [87]. A feature of pesticide suicides is that many patients reach hospital alive, providing opportunities for resuscitation. Data from China indicate that almost two thirds of pesticide deaths received some resuscitation that failed – improved training and resources for medical care may have prevented a number of these deaths[88]. Likewise, research and investments are needed to support community and facility based interventions aimed at reducing the incidence of self-harm overall. Such interventions need to be based upon policies and strategies that give due consideration to local culture, patterns of self-harm and institutional frameworks and not just a transfer of strategies from Europe or North America.

## Conclusion

Fatality estimates such as we have presented here under-represent the burden of disease because suicide has a long term impact on family, friends and work colleagues of the deceased. Furthermore, as we have shown, pesticide poisoning occurs predominantly in rural areas of low income countries, and the intense medical care required to treat pesticide poisoned patients may impact greatly on already stretched healthcare resources. By any measure, the burden of pesticide poisoning is large and merits urgent policy attention. However, attempts to prevent suicide in these areas and WHO's global suicide prevention strategy are largely modelled on findings from research and models of suicide prevention developed in the West. Research undertaken in low income communities – particularly in rural areas – is urgently required to provide the evidence base to underpin public health strategies for preventing pesticide suicides in these countries.

## Competing interests

DG and ME, are on the scientific advisory group of a Syngenta-funded study to assess the toxicity of a new formulation of paraquat, and have received travel expenses to attend research group meetings.

## Authors' contributions

DG had the idea for the study and developed the methodology and approaches to synthesis of the data jointly with ME, FK and MP. DG carried out the literature searching and wrote the first draft of the paper. All authors provided critical comments and were involved in re-drafting the manuscript. DG is guarantor.

## Pre-publication history

The pre-publication history for this paper can be accessed here:



## Supplementary Material

Additional file 1 Method used to estimate worldwide ventilator occupancy as a result of pesticide self-poisoning. This provides the calculations behind the estimates of ventilator use in paragraph 5 of the discussion section of the paper. Click here for file
