# Supplementary material for: The global distribution of fatal pesticide self-poisoning: Systematic review
Source: BMC Public Health. 2007 Dec 21;7:357. doi: 10.1186/1471-2458-7-357 (PMC2262093; doi:10.1186/1471-2458-7-357)
Supplement: Additional file 1 — Method used to estimate worldwide ventilator occupancy as a result of pesticide self-poisoning. This provides the calculations behind the estimates of ventilator use in paragraph 5 of the discussion section of the paper. [file 1471-2458-7-357-S1.doc]

# Additional File 1

Estimate of ITU person days spent on ventilators worldwide, (based on earlier estimate of 2,582,340 pesticide self-poisonings): A crude estimate of the number of patients intubated is (OP poisoning: 2/3 x 2,582,340 x 0.25 = 430,390; poisoning with other pesticides: 1/3 x 2,582,340 x 0.05 = 43,039; total 473,429). The length of time intubated will be [430,390 x 0.66 x 45 hrs] + [430,390 x 0.33 x 284 hrs] + [43,039 x 45 hrs] = 55,055,489 hrs of intubation – 6280 person years of ventilation. If the lower estimate of 1,291,170 episodes of pesticide self-poisoning is used, this equates to 3140 years of ventilation.
